# Care-‘less’: exploring the interface between child care and parental control in the context of child rights for workers in children’s homes in Ghana

**DOI:** 10.1186/s12914-018-0151-9

**Published:** 2018-02-20

**Authors:** Ernest Darkwah, Marguerite Daniel, Joana Salifu Yendork

**Affiliations:** 1 Department of Health Promotion and Development, University of Bergen, P.O. Box 7807, 5020 Bergen, Norway; 2 Department of Psychology, University of Ghana, P.O. Box LG84, Legon-Accra, Ghana

**Keywords:** Child rights, Children’s homes, Caregivers, Caregiving work, Parental control

## Abstract

**Background:**

This study explored how employed caregivers experience the interface between child care, parental control and child rights in the context of Children’s Homes in Ghana. The focus was on investigating caregiver perceptions of proper child care, their experiences with having to work with child rights principles and the implication of these for their relationships with the children and the care services they deliver.

**Methods:**

Adopting a qualitative approach with phenomenological design, data were collected from 41 caregivers in two children’s homes in Ghana using focus group discussions and in-depth interviews.

**Results:**

It emerged that caregivers experienced frustrations with perceived limitations that child rights principles place on their control over the children describing it as lessening and, at the same time, complicating the care services they provide.

**Conclusions:**

The findings suggest a need for a review of the implementation strategies of the child rights approach in that context. A re-organization of the children’s homes environment and re-orientation of caregivers and children regarding their relationship is also suggested.

**Electronic supplementary material:**

The online version of this article (10.1186/s12914-018-0151-9) contains supplementary material, which is available to authorized users.

## Background

Institutional spaces for the care and control of children in Ghana fall within an elaborate legal framework. In February, 1990, Ghana ratified the United Nations Convention on the Rights of the Child (UNCRC) and became the first country to do so. Soon afterwards, the government of Ghana followed this step with the formulation of the Children’s Act [1] and a plethora of other legislative and policy actions including the establishment in 2001 of a Women and Children’s Ministry, now Ministry of Gender, Children and Social Protection. The Child Reforms Initiative (CRI) was also introduced in 2005. According to the Department of Social Welfare (DSW), these were actions intended to demonstrate the commitment of the government of Ghana towards fulfilling its obligation of safeguarding the rights, safety and wellbeing of all Ghanaian children in whatever context they are found. Over the years, successive governments together with their international partners have continued in these efforts and designed child-rights oriented programmes seeking to implement these in all social spaces where children live their lives. Children’s Homes (CHs) - residential institutions that provide alternative care for children who have lost the care of their parents, are one example of such social spaces in Ghanaian society.

Described by the DSW as a ‘last resort’ for providing alternative care for children without parental care (CWPC) [2], CHs in Ghana take CWPC into residence and employ caregivers to act as their ‘parents’. The job of ‘*care*’ in this context implies that these caregivers assume ‘parental responsibilities’ for the children, providing emotional and psychological support, disciplinary training and overseeing their general wellbeing and development with material and financial support from their institutions and external donors. In performing their work roles as “parents”, caregivers are required by law and as a strict condition for funding from external, often western donors, to follow international child rights provisions backed by local legislations [2]. In particular, the provisions of the UNCRC are emphasized and used as the main guidelines for raising the children in this context.

### The UNCRC in the Ghanaian cultural context

As successful as the UNCRC has been regarding its usefulness in getting governments and policy makers to give more attention to children as a group of people who need special care and protection in all contexts, critics point out weaknesses in the convention’s ability to achieve positive impacts in different cultures. For example, Harris-Short [3] describes it as having been conceptualized on a legal system that is “founded on a ‘society of states’ in which the voices of the local and particular are effectively silenced”. In this sense, the argument has been that it does not proportionately reflect non-western ideologies of parenting and parent-child relations and therefore holds the potential to cause social tensions and confusions [4–6]. This is witnessed in Ghanaian society where the socio-cultural context is replete with parenting and child rearing norms, some of which are observed to be at odds with some of the provisions in the UNCRC. Twum-Danso [7] notes that socialization goals in local Ghanaian society require that children submit to the control of their parents or adult caregivers and certainly do as they are told without any questions. *Parental Control* - defined as the amount of supervision parents exercise, the decisions parents make about their children’s activities and friends, and the rules parents hold for their children [8] is a key ingredient in good parenting in Ghana and adults performing parental roles in whatever contexts within the society expect and are expected to maintain this form of control. Forms of child discipline such as corporal punishment are important characteristics of this traditional ideology of ‘good parental control’ [7, 9] and ‘*proper child care*’ is strongly associated with good ‘*parental control’*. This means that certain provisions in the UNCRC which, for example, seek to protect children from abuse by prohibiting actions like ‘beating to discipline’ have been a source of tension between local Ghanaian communities and child rights advocates [10]. Consequently, there have been difficulties implementing the international child rights provisions, or at least parts of it, in local Ghanaian communities where cultural and religious norms urge parents and guardians not to “spare the rod and spoil the child” [7, 9]. Thus, despite reported successes since child rights principles were introduced in Ghana, there have also been some challenges with researchers reporting tensions between parents and children over parents’ authority and children’s rights, separation of children from their parents in instances where authorities deem that such children’s rights have been significantly violated and, sometimes, alienation of children from their caregivers in instances where caregivers perceive the children they raise with UNCRC guidelines as belonging to foreigners [9, 11–13].

### Child rights in the Ghanaian Children’s home context

While the observed challenges with the implementation of child rights may be problematic for family social life in general Ghanaian society, its ramifications may be even more profound in specific social spaces like CHs where ‘hired parents’ provide care to vulnerable children. This is because there are already existing challenges in bonding between caregivers and residential children attributed to their natural non-blood relations and the socio-professional nature of the interaction between them [14–16]. The outcomes of these challenges have been reported to include caregiver-child relational problems, caregiver ambivalence, caregiver stress and burnout as well as general developmental deficiencies in residential children compared to their cohorts in normal family homes [17–21]. The observed negativities about the potential impacts of child rights provisions on adult-child relations in the Ghanaian context, therefore, raise questions about how the already existing challenges might be impacted by the introduction and subsequent dominance of these provisions in CHs. With traditional Ghanaian parenting ideology believing in parental control as central to the concept of ‘good care’, the question then becomes how caregivers perceive to provide adequate care or ‘good parenting’ for the children without having to exercise that kind of traditional control over them. Given that recent revelations from the institutional care environment for CWPC in Ghana have alleged relational tensions between the various players in that environment, [13, 22, 23], there is the need to investigate how the nexus between ‘Child care’ and ‘parental control’ plays out for workers in this occupational context where the UNCRC reigns supreme.

The present study investigated this nexus between child care and parental control by exploring the experiences of caregivers who play parenting roles in an environment dominated by child rights. The overall objective was to explore the interface between caregiver perceptions of proper child care, their experiences with having to work under the control of child rights and the implication of these for their relationships with the children and the care services they deliver. The purpose is to provide important scientific insights into the complexities of the residential care environment, and, perhaps, encourage debate about possible ways of engendering harmony in order to provide care workers with a healthy work environment that is supportive of their growth and service delivery.

### Research questions

Specific research questions were:

1. What are caregivers’ perceptions of proper ‘parental care’?
2. How do caregivers experience having to work with child rights principles?
3. What are caregiver’s perceptions of the impact of child rights on their ability to provide care?

## Methods

### Setting

The study was conducted in two regions of Ghana, West Africa. The regions have some of the country’s largest Children’s Homes with some run and funded by the government and others run and funded by private organizations. The CHs in these regions also differ in terms of organizational forms. Some are organized in forms reminiscent of boarding schools with children sleeping in large dormitory-style rooms and have shift-working caregivers, while others are organized in family home units with children living permanently with one caregiver in a small family home. CHs in these two regions were therefore purposefully selected in consideration of the differences in organizational form and funding sources as these would have implications for their operational practices. While the idea was not to compare understandings of child rights between government and private institutions, we sought to capture lived experiences of caregivers across the different contexts in order to generate a more balanced insight into the care work in CHs.

### Participants

Forty-one caregivers recruited from two Children’s Homes participated in the study. Caregivers were recruited for participation by first contacting them through visits to their institutions and directly asking for their participation after a thorough explanation of the research had been given. Only caregivers who volunteered and signed informed consent forms participated in the study. Participants consisted of ‘mothers’ and ‘fathers’ (core caregivers who had day-to-day contact with the children playing the role of ‘parents’). Although the primary purpose was to capture the experiences and perceptions of these core caregivers, it was also important to incorporate views and experiences of other workers in the residential care environment in order to develop deeper insights and better understand the totality of the context within which the phenomenon of care occurs. In this sense, institutional gatekeepers (directors and administrative staff), social workers, educational workers, former institutional children now working as volunteers and resident nurses were also contacted and subsequently recruited for participation. Table 1 presents demographic details of the participants:

**Table 1:** Detailed demographics of participants

Item	Category	Number
Sex	Male	8
	Female	33
Age Range	25–35	5
	36–45	5
	46–55	22
	56–58	9
Education	Post -Graduate	1
	Bachelor level	3
	Professional/Voc/Dip	11
	Middle school	26
Work Role	Manager/Director	2
	Mother	22
	Assistant Mother/Auntie	8
	Former institutional child/ Volunteer	3
	Resident Nurse	2
	Teacher	2
	Social Worker	2
Length of Service (in years)	0–10	6
	11–20	14
	21–30	13
	31–40	8
Marital status	Single	8
	Married	14
	Divorced	12
	Widowed	7

Source: Fieldwork data

### Research approach and data procedures

Given that the main focus of the study was on exploring subjective experiences of caregivers who work with CWPC in institutional environments, the general approach adopted here was qualitative. The epistemological and ontological grounds of relative truth and multiple realities upon which qualitative research approaches are founded see, [24, 25], made it imperative that this study adopts the approach in order to adequately address the research questions. Specifically, the Phenomenological design (both descriptive and interpretive) was used in order to be able to adequately capture the lived experiences of the participants regarding the phenomenon of care under child rights in the unique context where they work. Techniques for data collection therefore included focus group discussions and individual in-depth interviews.

#### Focus group discussions

Two focus group discussions were conducted (one in each institution) with 14 participants in total (8 in one and 6 in the other). Discussants were all mothers and ‘aunties’ aged between 45 and 58 years old with work experiences ranging from 3 years to 36 years. The mothers were caregivers in charge of home units within the institutional compounds while the aunties worked as assistants to the mothers. The first focus group discussion lasted 2 h and 3 min while the second lasted 2 h and 13 min.The focus group discussions allowed caregivers to express subjective as well as shared experiences, meaning making and perceptions about the phenomenon of care and the complexities of the care work in which they are involved. Themes that were put forward for discussion included: “What are your perceptions of proper parental care?”, “How does it feel having to work with child rights regulations in caring for these children?”, “How do child rights principles impact your care responsibilities?” and “How do child rights principles that dominate this environment affect your relationship with the children in your care?” (See Additional file 1). The lead author played the role of facilitator during the focus group discussions.

#### In-depth interviews

Individual, one-on-one interviews were conducted after the focus groups at times and locations convenient to each participant. The interviews were conducted to collect additional data to complement the data collected in the Focus groups. In all, 32 interviews involving mostly caregivers who did not participate in the focus groups were conducted across the two institutions. Eight (8) Focus group discussants however also took part in the interviews. These were individuals who the facilitator felt possibly had more to say but were held back in some way by the public situation in the focus groups. Themes put forward for discussion during the focus groups also formed the main questions during the interviews with the interviewer taking the opportunity to probe further into participant perceptions and experiences (See Additional file 1). The interviews lasted an average of one hour twenty-five minutes and were conducted in either English or Twi (local Ghanaian language) depending on the preference of the participant.

### Ethics

Ethical clearance was obtained from the Norwegian Social Sciences Data Services (NSD) in Norway where the lead author is a PhD student. The Department of Social Welfare of the Government of Ghana reviewed the research proposal and ethical clearance provided by our affiliated institution and deemed them sufficient enough for the research to be conducted. Permissions were also sought from the administrative offices of the institutions involved. All participants were individually approached and recruited after thorough explanations of the research had been given and informed consent obtained. For purposes of confidentiality, all interviews and focus group discussions were audio-recorded and the files kept in password-secured folders on the personal computer of the lead author.

### Data analysis

The collected data were translated, transcribed and coded. The coding process and subsequent analysis of the data were managed using Nvivo 10 software. For purposes of increasing the trustworthiness of the data, we enlisted the help of research colleagues who each independently coded the text after which all coders met and discussed the codes for consensus. In order to be able to better organize the data to identify emerging themes and the interconnectedness between main themes and sub-themes, we followed Attride-Sterlin’s Thematic Network Analysis (TNA) process for analyzing qualitative data [26]. We first identified and clustered codes expressing the same or similar ideas in the transcripts into key Basic Themes. Basic themes centering on the same or similar ideas were then clustered into Organizing Themes after which the organizing themes came under one Global Theme which captured the essence of the information obtained. This systematic, rigorous analysis process allowed for a better understanding of the underlying meanings by laying bare the linkages between themes and producing a better picture of participant experiences. Table 2 presents the TNA conducted:

**Table 2:** Thematic Network Analysis (TNA) of collected data

Codes	Basic Theme	Organizing Theme	Global Theme
…a good parent provides all children’s needs	Provide all children’s needs	Caregiver perceptions of proper parental care	Caregiver experiences of Child care and parental control under child rights
…you need to educate, support and provide for them			
…you have to be their life coach			
...you have to be someone they can trust in everything			
…You have to punish them when they go wrong	Correction by punishment		
…If you spare the rod you spoil your child			
…you can’t be a parent if you can’t correct your children			
…a good parent owns his children and trains them			
…a parent is like a leader, you need to be in charge	Maintain total control to guide them		
…they are children, they need your control for their own good			
…if you can’t control your children, then you can’t care for them			
…out of control children are an example of bad parenting			
… we have limited control over them because of child rights	Child rights take away parental control	Caregiver experiences with child rights and parental control	
…how can we parent them if we can’t control them			
… we can’t control them like our own children, it is against their rights			
…I agree with some of it, but it doesn’t allow us much control			
…child rights are not bad at all, they just don’t fit this environment	Child rights are Foreign and Incompatible with us		
…child rights are simply foreign			
…our children have rights here too, but not these foreign ones			
…it’s completely confusing			
…you feel like you can be arrested anytime in this job	Child rights threatens job loss and prosecution		
…I’ve had some of my colleagues sacked because of child rights			
…if you want to keep your job, just obey them			
…if you try to correct a child, you can be arrested for that			
…with all those rights, you can only do little for them	Lessened care under child rights	Child rights impacts on care duties and relationship with children	
…if you follow those rights strictly, you can only give less care			
…if I can’t punish my child, then I’m not giving enough parental care			
…I just leave them alone	Caregiver ambivalence		
…it is they (children) who will suffer here not me			
…we dint raise them, we just serve them			
…what do you do if the child does not listen to all your talk?			
…we know our limits, and they know our limits too	Reinforces professional rather than parental relationship		
…we are workers, not real parents so we just work			
…the child doesn’t see you as a parent, just a worker			
…the relationship is that of work not that of parenting			

## Results

Nine basic themes regarding the interplay between care and control within the framework of child rights for caregivers and how this influences their care responsibilities and relationships with the children in their care emerged from the data analysis. These were synthesized into three organizing themes and one global theme. In order to allow for clear and systematic presentation of our results, we arranged them in accordance with the organizing themes that emerged:

### Caregiver perceptions of proper parental care

In many regards, caregiver descriptions of what they perceived to be ‘proper’ parental care tended to be in tandem with global standards of parenting and seemed to concur with child rights advocates’ principles of how adults should handle children. Caregivers believed that a good parent should provide all resources including protection and guidance for children and be a beacon of trust for them:

*“…in my opinion, a good parent takes total responsibility for their children and provides everything they need…”(Mother, 51 years old)*

Another said:

*“..as a parent, you need to be someone your children can trust to both protect them and support them …you need to educate them, and provide for their needs… if you fail in these, you can’t call yourself a good parent”(Aunty, 38 years old)*

However, there were other perceptions that would be at odds or not in total agreement with child rights positions especially regarding control and punishment. For example, beliefs in control were persistent with some perceiving that control, punishment and care were inseparable in proper parenting. Some were of the view that ‘care’ is not possible without strong parental control:

*“…if you can’t control them, then how can you care for them?…they are children, they need your direction and care and you achieve that by guiding and correcting them, sometimes by necessary punishment…”(mother, 53 years old)*

A teacher said:

*“…You can’t be a parent if you can’t correct your children…you should be in control and punish them when they go wrong even if it means beating them because if you don’t beat them, they spoil…”(Teacher, 31 years old)*

At focus group discussions, belief in these forms of correction in parenting seemed unanimous and entrenched despite participants admitting to being trained to realize that such forms of correction might go against child rights.

*“…you might go against child rights if you hit them, but if that will correct them, then why not do it…”(Aunty, 43 years old)*

This raised the question of how they experienced child rights in their line of work and were coping with working under rules that were against their beliefs. It also brought up how they perceived themselves as “parents” of the children in their care.

### Experiences with child rights and parental control

Recounted experiences of working with child rights were mostly negative. Most caregivers felt that child rights were foreign and limited their ability to give ‘proper’ care to the children as they saw fit. This, to some brought a sense of guilt of failing in the duties that they signed up to perform:

*“…I feel that we should not allow foreigners to tell us how to raise our own children because we don’t have a say in how they raise theirs…those rules just don’t fit our environment…”(Aunty, 41 years old)*

*“…I came here to be a parent, but I can’t confidently say I am parenting these children…the child rights rules just tie your hands…”(Mother, 58 years old)*

*“…You can’t control them like your own children…it’s against their rights…you feel guilty when they do something wrong and all you do is just talk…they can listen or just ignore you…” (Mother, 55 years old)*

Some felt following the child rights rules that guide their work roles meant that they could only provide less care, while others recounted feeling threatened with prosecution and job loss by the child rights principles:

*“…Well, I do my best under the circumstances…I could do more for them, but thanks to those rules I can only do less care…I can’t train them like I do my own children…”(Mother, 47 year old)*

*“…You know, you could lose your job or even go to prison if you mess with those rules…and you were only trying to help the children…you just don’t feel comfortable at your workplace…”(Mother, 53 years old)*

Interestingly, higher institutional officers tended to hold positive perceptions of child rights and felt it was necessary to implement them. There was the acceptance that the principles are necessary for protecting children against abuse and giving them the best care:

*“…In my experience, I have come to see that those who made the rules had good reasons for making them, because things like beating could easily turn to abuse…it’s just unfortunate that over here it rather confuses people…”(Director, 54 years old)*

The differences in perceptions between core caregivers and higher institutional officers seemed to be causing tensions in that work context:

*“…I confronted one of them (core caregivers) on why she was making a child cook while the child should be sleeping after school. She told me that she has parented children some of who are older than me, so I’m not the one to tell her how to do her job…” (Social worker, 35 years old)*

During focus group discussion, this friction was highlighted when a caregiver quipped to the agreement of fellow discussants:

*“..All those officers know about parenting is from books they learnt at school, and those books are written by foreigners so all they know is foreign parenting…”(Aunty, 43 years old)*

Interviews with former institutional children who were now working as volunteers for their institutions indicated that they were unsure about their position on child rights:

*“…I don’t know really…sometimes it’s good, sometimes it’s bad….as a child I thought it was good, but now I feel our local ways should be allowed more because the children are getting spoilt…” (former inst. Child, 31 years old)*

### Child rights impacts on care duties and relationship with the children

Caregivers also recounted some positive and negative experiences regarding how child rights impacted their care duties and relationship with the children. For some, child rights made it possible for them to learn new ways of parenting and accept their work duties while others thought their workload is heavy because of child rights:

*“…I would say I have learnt new ways of parenting and I have accepted it and managed to calm down…I now carry out my work duties without complaint…”(Mother, 52 years old)*

*“…well, because we are afraid of breaking those child right things, we can’t ask the children to help us too much. You do much of the work yourself so the workload is heavy…In my own house, my children would help” (Mother, 54 years old)*

Caregiver accounts of how adherence to child rights principles affects their relationship with the children alluded to ambivalence, some selective attention and a sense of helplessness regarding how to handle the children. Others felt that the relationship was more of master-servant than parent-child:

*“…I don’t complaint at all. I just leave them alone. It is they who will spoil and suffer in the future, not me or my own children…”(Aunty, 43 years old)*

*“…Well, those child rights help…you can see that some of the children are happy and respond to you positively when you use the rights approach. But there are others who you need our local approach to get them to behave…but since we can’t do that, we just pay attention to those who listen to us and leave the rest alone…”(Mother, 56 years old)*

*“…(laughs) I don’t believe we parent them even though that is supposed to be our job…we just serve them. They are the masters, we are the servants and they know that so they don’t respect us…”(Mother, 55 years old)*

Thus our results show that caregivers perceived proper parenting to significantly include high parental control the lack of which made them experience the CWPC care job in the CH context negatively. Perceptions were that parenting roles in the CH context were complicated by the use of child rights approaches. There were reports of tensions between core caregivers and institutional officers as a result of child rights and a negative impact of child rights on caregiver-child relationships.

## Discussion

The present study explored caregivers’ conceptualization of children’s rights and the impact on their care responsibilities in two residential settings in Ghana. To achieve this broad goal, three research questions were explored. The first was to examine caregivers’ perceptions of what constitute good parental care. Participants’ reports revealed two diverse views that support and contradict the international principles and goals of children’s rights. Some participants reported that good parental care consists of the provision of material resources, guidance and protection for children. These findings are consistent with previous research in Ghana [27] and global perceptions of good parental care [28, 29] which highlights the need for parents to be dependable and provide for children’s needs in order to ensure survival. The emergence of these perceptions suggests that although the caregivers might not fully understand or accept aspects of the UNCRC principles, their perceptions of parental role align with the core aims of UNCRC, which is to enhance children’s welfare.

In contrast, there were reports of parental control and use of punishment as being key components of proper parenting. This perception contradicts UNCRC’s core aims of providing children with the right to freedom of expression and protecting them from all forms of abuse and punishment. However, the findings reflect the Ghanaian cultural notion of parenting in which parents assume control over all affairs pertaining to children and children are expected to yield to the demands and authority of parents [8, 10]. Additionally, the emphasis on corporal/physical punishment also aligns with acceptable cultural methods of child-rearing processes by both caregivers and children in Ghana [9, 10]. Participants’ notion of the constituent of good parenting thus highlights the influence of the broader socio-cultural context in shaping individuals’ views of parenting roles [30]. The endorsement of physical punishement as constituent of good parenting corresponds with the group differences hypothesis of parenting effects on child outcomes [31]. This hypothesis proposes that development occurs within a cultural context and the individual’s developmental processes reflect an adaptive response to environmental demands. Thus, although the use of physical punishement is associated with negative outcomes in some cultures, especially where its use is frowned upon (e.g., European, American culture), its use is not associated with adverse outcomes in other contexts where such practices are normative (e.g., African-American and African cultures) [31, 32]. Indeed our findings, as shown in caregivers responses, confirm suggestions by Deater-Deckard et al. [31] that *not* using physical punishment is seen in some cultures as neglect on the part of the parent or parenting figure.

The disparity between principles of children’s rights and notions of “proper parenting” in the present sample therefore underscore the concerns raised by critics of the provisions in UNCRC [4–6] and supports previous research that has highlighted similar challenges of implementing the UNCRC in the Ghanaian socio-cultural context [33]. It confirms that there are aspects of the children’s rights principles that do not match with the Ghanaian cultural processes of child rearing. Such disparity is likely to create internal tension in caregivers over what should take precedence in their caregiving practices, their socialized beliefs or job-required rules of parenting. The internal tensions in turn could also precipitate work role confusion as the caregivers complain about being confused regarding how they are expected to parent under such foreign rules. The bigger problem is that, in situations where individuals are only acting in loco parentis of children, as in the case of children’s home, there is the potential for neglect of parenting duties, especially with discipline (in fear of not violating child right rules) and emotional support (due to conflicts in child-caregiver relationship). Hence, parent-child relationship could become mechanistic in nature.

Our second research question was to explore caregivers’ experiences of working with child rights principles. Overall, we found conflicting accounts when core caregivers’ reports were compared with that of the higher institutional personnel. Core caregivers mostly reported negative experiences working with child rights principles with many reporting that child rights were incompatible with known cultural methods of parental care and further limited their ability to offer good parenting to children. These perceptions confirm earlier reports in which researchers have found that the notion of child rights are not easily accepted in Sub-Saharan African cultures and seen as an instrument that distorts the way their society cares for its children [34, 35]. The findings suggest lack of understanding and appreciation for the essence of children’s right principles. For these caregivers, incorporation of children’s rights principles seems to be an invasion of their cultural socialization processes of parenting by foreign principles. Given their perceptions, it appears that children’s rights principles were not perceived as tools for improving children’s well-being, but a means of control by foreign agencies. Feelings of apprehension over prosecution and job loss also show that these caregivers have attempted to adapt children’s rights principles into their caregiving practices only to avoid personal adverse consequences (such as job loss) but have no faith in the essence of child rights principles in caregiving. Instead, the emotional attachment that should accompany caregiving is lost for these caregivers. The goal of improving children’s welfare with children’s rights is replaced with saving their career.

Despite primary caregivers’ negative experiences with children’s rights, higher institutional officers held positive views of child rights and expressed the need for their implementation in order to protect children from harmful caregiving. The differences between the two categories of professionals could have resulted from the differences in job roles, in that whereas the higher institutional professionals focus on policy administration and answer directly to donors and government, the core caregivers have direct contact and manage the day-to-day affairs of the children and thus feel the practical impact of implementing the child rights principles. These findings contradict previous research in Ghana [27] that found similar perceptions of children’s rights when views of two professionals were compared. Additionally, the challenges reported by the present participants are different from those in Manful and Manful’s study who mainly reported lack of information and resources to implement children’s rights principles. From our findings, the current challenge has to do with acceptance and adaptation of a “foreign” concept of child-rearing. This is problematic in that the perceived lack of convergence between caregivers’ socio-cultural norms of child care and “foreign” principles impedes caregivers’ ability to establish emotional relationships vital for good socio-emotional development in children. What is also worth highlighting is the tension that has been created between core caregivers and higher institutional officers due to differences in perception about the relevance of children’s rights policies. This tension could harm co-operation and employee relations inside the children’s home and could negatively impact on caregiving.

The third research question focused on exploring caregivers’ perceptions of the impact of child rights on their ability to provide care. Both negative and positive accounts were given. On the positive side, some participants reported that child rights principles provided new parenting skills. On the other hand, others perceived child rights as a burden that increased workload and negatively affected the caregiver-children relationship. Due to the perceived limitations and burden that come with child rights, caregivers reported feeling helpless and unable to properly offer care but rather being forced to neglect their responsibilities of discipline. This confirms previous research [35–37] that has questioned the ability of the children’s rights convention to improve children’s welfare across different cultures. Participants’ reports show that caregivers who are tasked to practically execute the principles of children rights are rather expressing more harmful effects. Instead of improving children’s welfare, it is rather impeding child care, thus, highlighting a gap between the intent and practical applications of UNCRC.

Our findings support Ame, Apt and Agbenyega (2011) and Porter et al., (2011) in arguing that there is the need for intensification of education on child rights in Ghana. Particularly, our findings imply that calls made by researchers such as Embleton et al., (2014) regarding increasing need for education of staff in residential institutions on the principles of children’s rights in order to enhance their understanding and acceptance are in the right order. Improved understanding may increase acceptance, reduce anxieties and promote pleasant relationships between caregivers and children. There is also the need for training aimed at equipping caregivers with practical skills essential for incorporating children’s rights principles into caregiving practices. Such training should entail skills for problem solving, behavior modification techniques, improving communication, and stress management. These techniques would improve caregivers’ ability to handle relationship problems between themselves and the children as well as appropriate strategies for handling children’s behavioural problems rather than resorting to use of physical punishment. Additionally, guidelines for building positive relationships as well as teaching life skills, autonomy and independence to children will help promote understanding and cordial relationship between caregivers and children. Engagement with local caregivers for their views, conceptualization of, and experiences with, children’s rights is also encouraged. Such engagement would provide an entry point for discussion on ways to develop policies and programmes that would be culturally relevant for promoting children’s welfare. Furthermore, there is the need for review of UNCRC in terms of its applicability in the Ghanaian context in conjunction with infrastructure and social-cultural norms and practices that impede the successful implementation of children’s right.

The above implication accepts that UNCRC is universally applicable and that people (i.e. caregivers) have to change to accept children’s rights and ensure they are appropriately operationalized. However, it is worth questioning whether it might be possible to reformulate some children’s rights to be more culturally inclusive. The African Charter on the Rights and Welfare of the Child (ACRWC) [38], published the year after the UNCRC, gives possible leads on this question. Many of the articles in ACRWC are very similar to those in UNCRC, however, two significant differences will be highlighted here. Firstly, in the UNCRC it is not stated who has primary responsibility for the child whereas the ACRWC states clearly in Article 20.1 that parents shall have the primary responsibility for the upbringing of the child and in Article 20.2 that the State shall assist parents in case of need. Secondly, UNCRC has nothing about the *responsibilities* of the child, whereas Article 31 of the ACRWC it states that it is the responsibility of the child, among others, to respect his/her parents and assist them in case of need, and to preserve and strengthen African cultural values. Including these two aspects in the UNCRC might make children’s rights more acceptable to caregivers such as those in our study. It is interesting that although Ghana was the first country to ratify the UNCRC, it was only in 1997 that it signed the ACRWC and only in 2005 that it ratified it(African Commission on Human and People’s Rights, 2017), much later than many other African countries. The explanation for this may be found in Magesan [39] argument that simply ratifying human rights treaties (as opposed to real changes in human rights behaviours) increases aid received per person both in the short and the long run.

### Relexivity

The qualitative data collection techniques used in this study imply that the lead author who was responsible for data collection could have influenced how participants responded to the research. Considering that the lead author is a Ghanaian of high education relative to the educational status of the participants, and living abroad (which increases a person’s social status in local Ghanaian communities) it was possible that power differentials could influence the researcher-participant relationship in a way that could put the researcher in a position of power and the participant feeling obliged to respond in ways that may please the researcher as suggested by Råheim et al. [40]. In view of this we took specific steps to try to create a welcoming, non-authoritarian, non-threatening and open environment as a way to encourage openness and authenticity. Prior to data collection the lead author paid frequent visits to the institutions to interact informally with caregivers and children, and through this, build rapport with them to try to reduce the potential power distance before the actual focus groups and interviews began. Over the course of the two months of data collection, the lead author was careful to explain himself as ignorant in that field of work seeking to listen to and understand the experiences of caregivers who are experts in the care work in that context. It was observed that over time, the caregivers had become used to the researcher with some who had previously declined participation turning round to ask if they could participate, in which case they were included. We also left participants to decide times and places that would be convenient for them for the focus group discussions and individual interviews with the interviews in particular taking place in participants’ homes inside their organizations. Nontheless the lead author was present during focus group discussions acting as a facilitator. It is therefore possible that his presence could have influenced the nature of participant responses. In order to reduce the impact that such possible influence could have on the data gathered, we held all interviews after focus group discussions with some of the focus group participants also taking part in the interviews. This was intended to provide a second chance to participants to confirm their stories, provide more detail or make modification in a private environment.

### Limitations and directions for future research

First, the present study used large residential institutions in Ghana. Hence the findings might not fully reflect experiences of caregivers in smaller residential care settings. Future research should consider including caregivers in smaller children’s homes and those in the rural areas in order to broaden information on caregivers’ experiences of children’s rights in residential settings. Second, our study purposefully explored caregivers’ experiences of their jobs and the rules governing work in the workplace. Thus only views of caregivers but not children were explored in the present study. Exploring residential children’s perceptions and experiences with child rights could provide relevant insight into the direct impact of the application of UNCRC on children’s well-being in settings such as the children’s homes. It could also shed light on areas of divergence in experiences of caregivers and children as well as provide ideas on ways to amend the challenges with implementation of child rights. Hence, future research should explore children’s experiences with child rights. Third, only residential care settings were used for the present study. However, children’s rights are applied in other child-related professions in other settings. Another area of research would be to explore the views of caregivers in non-residential setting such as families, schools and hospitals in order to generate a basis for comparison with residential settings to ascertain how child rights are conceptualized and applied in these settings. Fourth, in children’s homes, caregivers are expected to go beyond the provision of professional care to include parental care to children. This expectation disregards professional issues relating to staff regulation, pay, recruitment, conditions of service and worker’s rights that may interfere with caregivers’ ability to act as parents. Such expectations also neglect potential trauma caused by parental loss and substitution of parents by caregivers on children’s well-being. Future research could explore issues of professional care versus parenting and how they impact on caregivers and children in chilren’s homes. Lastly, given the disparity between child rights principles and Ghanaian cultural norms of parenting that led to tensions in the children’s homes care environment, the field would benefit from studies that explore the tensions between management expectations and realities of caregivers where the joint from child rights to real care work take place. Despite the limitations of the present study, the findings have shed light on how children’s rights are perceived, the experiences of professionals working with the principles as well as the impact of the UNCRC on caregiving and children’s welfare in a residential setting.

## Conclusion

We set out to explore employed caregivers’ experiences regarding the interface between child care, parental control and child rights in residential care institutions for children without parental care in Ghana. We found that some caregivers perceived child rights principles as a form of *control* that limits their ability to deliver quality care services due to its prohibition of absolute parental control. This perception stems from the wide deviation of the child rights principles from Ghanaian cultural norms of child care. Hence, children’s rights principles are viewed as irrelevant and a hindrance to “proper” child care. On the other hand, other caregivers perceived child rights to offer an opportunity to gain new parenting skills that are different from the cultural norms of child rearing. To these caregivers, the new skills have helped enhanced their caregiving role to the children in their care. In this regard, views on child rights was congruent with participants’ perception of ‘proper’ parenting.

Our findings also showed that implementation of UNCRC has occurred at the institutional level albeit laden with several challenges that negatively affect the realization of its overall benefits in the Ghanaian context. Our study shows that the intent of child rights policies is not fully understood and not well accepted by employed caregivers tasked to execute the principles in children’s homes. As a result, rather than improving children’s well-fare, negative impacts have been reported. Additionally, for some caregivers, the presence of the UNCRC appears to have reduced the emotional component and spontaneity that naturally goes with child care and replaced with a ‘mechanistic’ version where caregivers see child care as a job and where rules are obeyed to prevent job loss. The convention which was established to frame the good care of children is thus perceived as doing the opposite in the context of children’s homes in Ghana.

## Additional file

Additional file 1: Thematic Interview Guide. This document contains the specific thematic questions put forward for discussion during Focus Group Discussions and Individual in-depth interviews for this study. (DOCX 15 kb)

## Abbreviations

CH: Children’s Home
CRI: Child Reforms Initiative
CWPC: Children without parental care
DSW: Department of Social Welfare
TNA: Thematic Network Analysis
UNCRC: United Nations Convention on the rights of the Child
