# Supplementary material for: Care-‘less’: exploring the interface between child care and parental control in the context of child rights for workers in children’s homes in Ghana
Source: BMC Int Health Hum Rights. 2018 Feb 20;18:13. doi: 10.1186/s12914-018-0151-9 (PMC5819201; doi:10.1186/s12914-018-0151-9)
Supplement: Supplementary file 1 — Thematic Interview Guide. This document contains the specific thematic questions put forward for discussion during Focus Group Discussions and Individual in-depth interviews for this study. (DOCX 15 kb) [file 12914_2018_151_MOESM1_ESM.docx]

**THEMATIC INTERVIEW GUIDE**

(To be used for FGDs and Individual interviews)

**Title: Care-‘Less’: Exploring the interface between child care and parental control in the context of child rights for workers in Children’s Homes in Ghana.**

1. **Demographic Questions**

- Sex:
- Age:
- Education level:
- Work role:
- Length of service:
- Marital status:

1. **Main thematic areas**
2. What are your perceptions of proper parental care?
3. How does it feel having to work with child rights regulations in caring for these children?
4. How do child rights principles impact your care responsibilities?
5. How do child rights principles that dominate this environment affect your relationship with the children in your care?

**Notes:**

• Interviewer to probe for more clarifications depending on participant responses

• Look out for comments on ***parental control*** and probe further
